# GAIT SPEED MODERATES EFFICACY OF HOME-BASED EXERCISE ON FALLS IN OLDER ADULTS WITH PREVIOUS FALL

**DOI:** 10.1093/geroni/igae098.0361

**Published:** 2024-12-31

**Authors:** Jordyn Rice, Ryan Falck, Jennifer Davis, Larry Dian, Kenneth Madden, Wendy Cook, Karim Miran-Khan, Teresa Liu-Ambrose

**Affiliations:** University of British Columbia, Vancouver, British Columbia, Canada; University of British Columbia, Vancouver, British Columbia, Canada; University of British Columbia, Vancouver, British Columbia, Canada; University of British Columbia, Vancouver, British Columbia, Canada; University of British Columbia, Vancouver, British Columbia, Canada; University of British Columbia, Vancouver, British Columbia, Canada; University of British Columbia, Vancouver, British Columbia, Canada; University of British Columbia, Vancouver, British Columbia, Canada

## Abstract

Exercise is an evidence-based strategy for preventing falls. However, its efficacy may vary based on individual characteristics, like gait speed. We performed a secondary analysis of a randomized controlled trial, to assess if baseline gait speed moderated the effects of an exercise program on subsequent falls among older adults. Community-dwelling adults >70 years who had fallen within the previous 12 months were randomized to either 12 months of home-based exercise (EX; n=172) or standard of care (SC; n=172). For this study, we examined intervention effects on fall rates at 6 and 12 months stratified by baseline gait speed (slow < 0.80m/s or normal >0.80 m/s) using negative binomial regressions. We also investigated whether baseline gait speed moderated the intervention effects on mobility and cognitive function using linear mixed modeling. At baseline, 134 participants had slow (EX=70; SC=64) and 210 had normal (EX=102; SC=108) gait speed. For participants with slow gait speed, EX reduced fall rates by 44% at 6 months (IRR=0.56; 95% CI:[0.33, 0.95]; p= 0.03) compared with SC; for participants with normal gait speed, there was no significant effect of exercise on fall rates at 6 or 12 months. Gait speed moderated intervention effects; EX participants with slow gait improved: 1) Timed Up-and-Go at 6 months (estimated mean difference= -4.05; 95% CI:[-6.82, -1.27]; p= 0.00); and 2) Digit Symbol Substitution Test at 12 months (estimated mean difference= 2.51; 95% CI:[0.81, 4.21]; p=0.00). Our study suggests older adults with slow gait speed may be a target population for exercise-based falls prevention.

